# Sudden Sensorineural Hearing Loss in Patients Aged from 15 to 40 Years

**DOI:** 10.3390/jcm13113303

**Published:** 2024-06-04

**Authors:** Mirko Aldè, Umberto Ambrosetti, Gioia Piatti, Camilla Romanini, Eliana Filipponi, Federica Di Berardino, Diego Zanetti, Lorenzo Pignataro, Giovanna Cantarella, Stefania Barozzi

**Affiliations:** 1 Department of Clinical Sciences and Community Health, University of Milan, 20122 Milan, Italy; umberto.ambrosetti@unimi.it (U.A.); camilla.romanini@studenti.unimi.it (C.R.); eliana.filipponi@unimi.it (E.F.); federica.diberardino@unimi.it (F.D.B.); diego.zanetti.bs@gmail.com (D.Z.); lorenzo.pignataro@unimi.it (L.P.); giovanna.cantarella@unimi.it (G.C.); stefania.barozzi@unimi.it (S.B.); 2 Audiology Unit, Department of Specialist Surgical Sciences, Fondazione IRCCS Ca’ Granda Ospedale Maggiore Policlinico, 20122 Milan, Italy; 3 Department of Pathophysiology and Transplantation, University of Milan, 20122 Milan, Italy; gioia.piatti@unimi.it; 4 Unit of Bronchopneumology, Fondazione IRCCS Ca’ Granda Ospedale Maggiore Policlinico, 20122 Milan, Italy; 5 Otolaryngology Unit, Department of Specialist Surgical Sciences, Fondazione IRCCS Ca’ Granda Ospedale Maggiore Policlinico, 20122 Milan, Italy

**Keywords:** sudden sensorineural hearing loss, young adults, cochlear endolymphatic hydrops, infectious diseases, autoimmune diseases, vascular diseases

## Abstract

**Objectives:** The purpose of this study was to investigate the hearing characteristics and causes of sudden sensorineural hearing loss (SSNHL) in patients aged from 15 to 40 years, focusing on audiological outcomes one year after the diagnosis. **Methods:** The medical records of individuals with SSNHL who were referred to our tertiary-level audiologic center were reviewed. All patients had undergone comprehensive diagnostic evaluations, including high-resolution 3D-FLAIR delayed magnetic resonance imaging (MRI), cone beam computed tomography (CBCT), and screening for coagulation, infectious, and autoimmune diseases. **Results:** Overall, 56 patients (mean age 28.1 ± 7.6 years) were included in the study. The hearing threshold in the affected ear improved significantly from 56.0 ± 18.0 dB at the diagnosis to 46.9 ± 22.3 dB after one year (*p* = 0.02). The degree of hearing loss, audiometric configurations, hearing improvements, and adherence to hearing treatments showed considerable variability among patients. Aural fullness, tinnitus, and hyperacusis were the predominant symptoms associated with SSNHL, and their prevalence decreased significantly over time. The diagnostic protocol led to the identification of the specific cause of SSNHL in 75% (42/56) of patients. The known etiology was found to be otological (39.3%), infectious (21.4%), autoimmune (7.1%), vascular (5.4%), or neoplastic (1.8%). In particular, Menière’s disease (n = 12), isolated cochlear endolymphatic hydrops (n = 6), HSV-1 (n = 5), and EBV (n = 4) infections were the most frequent causes of SSNHL. **Conclusions:** The identification of the specific etiology of SSNHL may facilitate a more personalized approach to management and treatment.

## 1. Introduction

Sudden sensorineural hearing loss (SSNHL) is defined as a sensorineural hearing loss (HL) ≥ 30 dB affecting ≥ 3 consecutive audiometric frequencies within a 3-day time frame [1,2]. It is recognized as a medical emergency requiring swift identification and treatment [3,4], although up to two-thirds of patients may experience spontaneous recovery of the hearing threshold (HT), especially within the first 2 weeks [2,5].

In most cases, SSNHL occurs in only one ear and can be associated with other bothersome symptoms, such as tinnitus, aural fullness, and vertigo [1,2].

The incidence of SSNHL increases with age, ranging from 1.2 per 100,000 for patients younger than 9 years to 77 per 100,000 for patients older than 65 years [6,7]. Possible causes of SSNHL include infectious, otological, vascular, autoimmune, metabolic, and neoplastic disorders; however, the underlying etiology often cannot be identified, and therefore, SSNHL is considered idiopathic [1,2,3,8]. All patients with new-onset aural fullness should be evaluated as soon as possible to distinguish sensorineural HL from conductive HL [2,4]. Otoscopic examination of the ear is necessary to exclude obstructing earwax, foreign bodies, or pathologies of the external or middle ear (e.g., otitis external and otitis media), while pure-tone audiometry is essential to confirm the diagnosis of SSNHL and define audiometric configuration and degree of HL [2,4,8]. Despite the limitations of existing evidence on the efficacy of medical therapy, corticosteroids, if administered within 14 days of symptom onset, are considered a viable treatment choice for patients diagnosed with SSNHL [2,5]. Hyperbaric oxygen (HBO2) may also be a potential treatment option within the first 14 days after the initial diagnosis of SSNHL, or up to 1 month as salvage therapy, in combination with steroids [2,8]. In addition, in case of incomplete recovery of the HT, intratympanic steroids may be proposed as salvage therapy 2 to 6 weeks after the onset of SSNHL [2]. Conversely, the use of antivirals, vasodilators, thrombolytics, or vasoactive drugs is not routinely recommended in patients with SSNHL because of the lack of proven efficacy [2]. However, it is important to note that treatment strategies can vary greatly depending on the specifics of each patient, such as the underlying cause of SSNHL, the patient’s overall health status, and personal response to medications [1,2].

The purpose of the present study was to assess in detail the hearing characteristics and causes of SSNHL in patients aged from 15 to 40 years who attended a tertiary-level audiologic center, focusing on audiological outcomes one year after the diagnosis.

## 2. Materials and Methods

### 2.1. Data Collection

In the present study, the medical records of patients aged from 15 to 40 years who were referred to our audiology clinic at the Foundation IRCCS Ca’ Granda Ospedale Maggiore Policlinico of Milan (a tertiary referral hospital in Italy) for suspected SSNHL during the period between 1 January 2018 and 30 April 2023 were examined. The study included all patients 15 through 40 years of age who were diagnosed with SSNHL and underwent the following procedures:

- Thorough collection of medical history;
- Oral corticosteroids in a tapering regimen (started within one week from the diagnosis of SSNHL): 60 mg once daily for 7 days, 40 mg for 3 days, 20 mg for 2 days, and 10 mg for 2 days;
- HBO2 treatment (started within one week from the diagnosis of SSNHL): more than 8 sessions (up to a maximum of 24) for 6 days per week of 85 min each with inhalation of pure oxygen at a pressure of 2.5 atmospheres absolute (AT);
- Otomicroscopy, with removal of earwax if present (before performing any audiometric evaluation);
- Pure-tone audiometry at frequencies from 125 Hz to 8000 Hz (within 3 days from the onset of symptoms (T0), immediately after the treatment with oral corticosteroids and HBO2 (T1), and 12 months after the diagnosis of SSNHL (T12);
- Tympanometry and stapedial reflex measurements (at T0, T1, and T12);
- Thrombophilia screening (within 2 weeks from the diagnosis of SSNHL): complete blood count, protein C, antithrombin III, protein S, activated partial thromboplastin time (aPTT), prothrombin time (PT), homocysteine, folic acid, prothrombin gene mutation (G20210A), factor V Leiden mutation, anti-β-2-glycoprotein-1 antibodies (IgG and IgM), lupus anticoagulant, and anti-cardiolipin antibodies (IgG and IgM);
- Autoimmune screening (within 2 weeks from the diagnosis of SSNHL): antinuclear antibodies (ANA), anti-cyclic citrullinated peptide (Anti-CCP) antibodies, rheumatoid factor (RF), anti-double-stranded DNA (Anti-dsDNA) antibodies, anti-extractable nuclear antigen (anti-ENA), antineutrophil cytoplasmic antibodies (ANCA), complement components (C3,C4), immunoglobulins (IgA, IgG, IgM), ferritin, C-reactive protein (CRP), and erythrocyte sedimentation rate (ESR);
- Infectious disease screening (within 2 weeks from the diagnosis of SSNHL): anti-Herpes simplex virus type 1 (HSV-1) and type 2 (HSV-2) IgM and IgG, anti-Cytomegalovirus (CMV) IgM and IgG, anti-CMV IgG avidity, anti-viral capsid antigen (VCA) IgM and IgG, anti-early antigen (EA) IgG, anti-Epstein–Barr nuclear antigen (EBNA) IgG, anti-SARS-CoV-2 (severe acute respiratory syndrome coronavirus 2) IgG and IgM, and anti-Borrelia burgdorferi IgG and IgM;
- Kidney, thyroid, and liver function tests (within 2 weeks from the diagnosis of SSNHL): complete urinalysis, glomerular filtration rate (GFR), creatinine, free-triiodothyronine (FT3), free-thyroxine (FT4), thyroid stimulating hormone (TSH), glycated hemoglobin (HbA1c), alanine transaminase (ALT), aspartate transaminase (AST), gamma-glutamyl transferase (GGT), and alkaline phosphatase (ALP);
- High-resolution magnetic resonance imaging (MRI) of the brain (3 Tesla scanner) including three-dimensional fluid-attenuated inversion recovery (3D-FLAIR) sequences acquired 10 min and 4 h after gadolinium injection (within 2 weeks from the diagnosis of SSNHL);
- High-resolution cone beam computed tomography (CBCT) of the ear (within 2 weeks from the diagnosis of SSNHL);
- Vestibular examination (at T0): Romberg test, Fukuda stepping test, visual/oculomotor assessment (convergence, saccades, smooth pursuit, and static visual acuity), observation of spontaneous nystagmus with Frenzel glasses, Dix–Hallpike maneuver, Pagnini–McClure maneuver, head shaking test (HST), dynamic visual acuity test, and video head impulse test (v-HIT);
- Genetic testing by next-generation sequencing (NGS) technology (with a total of 101 genes analyzed) [9].

The study established specific exclusion criteria, namely patients with the following:

- Any unilateral or bilateral HL prior to the diagnosis of SSNHL;
- Any disorders affecting the external or middle ear;
- Any ear surgery during the lifetime;
- Conductive or mixed HL and type B tympanogram.

In the present study, the following hearing variables were evaluated in detail: age at diagnosis, side affected by HL (at T0 and T12), hearing status (at T0 and T12), presence of hearing fluctuations over months, audiometric configuration (at T0 and T12), hearing changes between T12 and T0, and hearing treatment at T12. The assessment of hearing recovery one year after the diagnosis of SSNHL (at T12) was conducted using Siegel’s criteria, which are outlined as follows [8,10]:

- Complete recovery: if the final HT is <25 dB, irrespective of the extent of the gain;
- Partial recovery: if the final HT is 25–45 dB, with a gain >15 dB;
- Slight recovery: if the final HT is >45 dB, with a gain >15 dB;
- No improvement: if the final HT is >75 dB or the gain is <15 dB.

Moreover, at T0 and T12, the following symptoms were investigated through targeted questions asked during the audiologic examinations: vertigo (erroneous sensation of self or environmental motion perceived as spinning movement), disequilibrium (lack of stability, particularly during walking), tinnitus (hearing a sound when no external sound is present), aural fullness (sensation of increased pressure in the ear), otalgia (feeling of pain in the ear), and hyperacusis (reduced tolerance for ordinary sounds) [11,12]. If abnormalities were detected in the diagnostic tests, the patient was then referred to the most appropriate specialist physician (e.g., to the infectious disease specialist in the event of IgM positivity on the infectious screening, or to a hematologist if one or more values in the thrombophilia screening were found to be pathological) for further specific investigations and treatments.

The classification of HL was based on the pure-tone averages at frequencies 500 Hz, 1000 Hz, 2000 Hz, and 4000 Hz (PTA-4) [13]: mild HL (PTA-4 = 26–40 dB HL), moderate HL (PTA-4 = 41–60 dB HL), severe HL (PTA-4 = 61–80 dB HL), and profound HL (PTA-4 > 80 dB HL).

This retrospective study was conducted in strict observance of the World Medical Association’s Declaration of Helsinki and garnered the requisite approval from the local ethics committee.

### 2.2. Statistical Analysis

Statistical assessments were executed employing Stata 17 software (StataCorp., College Station, TX, USA, 2021). The Fisher’s exact test was utilized for the analysis of categorical variables. When the *p*-value fell below 0.05, it was interpreted as an indication of statistical significance in the results.

## 3. Results

The study included a total of 56 patients (17 males and 39 females) diagnosed with SSNHL who fulfilled the inclusion criteria. The mean age at the diagnosis was 28.1 ± 7.6 years (range 15–40).

**Figure 1:**
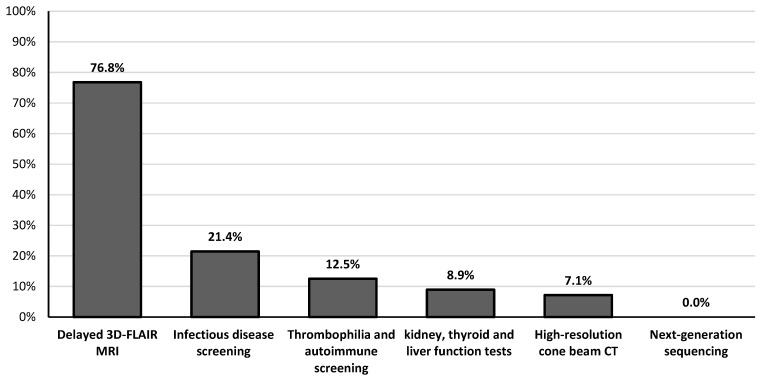
Frequency of detection of pathological findings among the different diagnostic assessments performed on patients with sudden sensorineural hearing loss.

Table 1 summarizes the main hearing characteristics of patients with SSNHL at the time of initial diagnosis (T0) and one year later (T12). Seven (12.5%) patients showed HT deterioration (> 10 dB HL) in the affected ear at the T12 assessment. According to Siegel’s criteria, complete, partial, and slight HT improvements were observed in 19.6% (11/56), 17.9% (10/56), and 3.6% (2/56) of patients, respectively. Overall, the HT in the affected ear improved significantly from 56.0 ± 18.0 dB (range 32.5–100) at diagnosis (T0) to 46.9 ± 22.3 dB (range 10–100) after one year (T12) (*p* = 0.02). In contrast, comparing the HT immediately after treatment with oral corticosteroids and HBO2 (46.0 ± 20.2 dB, range 15–100) with the HT at T12, no significant differences were observed (*p* = 0.9). No patients underwent intratympanic steroid injection in the 6 weeks after the diagnosis of SSNHL. The patient with bilateral SSNHL showed no improvement in HT over time, while the subjects with unilateral SSNHL had no hearing deterioration in the healthy contralateral ear. Findings from high-resolution MRI and CBCT as well as vestibular examinations are detailed in Table 2. Eight patients experienced labyrinthitis (seven unilaterally and one bilaterally), implying that SSNHL was associated with acute peripheral vestibular hypofunction (Table 2). The causes of labyrinthitis were HSV-1 infection (n = 2), Epstein–Barr virus (EBV) infection (n = 1), SARS-CoV-2 infection (n = 1), Cogan’s syndrome (n = 1), and idiopathic (n = 3). Clinical signs of acute unilateral peripheral vestibular hypofunction were elicited upon bedside examination (lateral inclination of the body toward the affected site during the Romberg test, deviation > 45° toward the side of weakness during the Fukuda stepping test, spontaneous horizontal–torsional nystagmus beating away from the side of the lesion, ≥3 beats of nystagmus toward the healthy side on the HST, >2 line differences between static and dynamic visual acuity, normal convergence, smooth pursuit and saccades, and negative Dix–Hallpike and Pagnini–McClure maneuvers) and confirmed by v-HIT (presence of catch-up saccades and reduced vestibulo-ocular reflex gain on the side of the lesion). Bilateral peripheral vestibular hypofunction was characterized by imbalance with absence of vertigo, while v-HIT revealed corrective saccades and reduced vestibulo-ocular reflex gain on both sides. The most common audiological symptoms initially associated with SSNHL were aural fullness and tinnitus, reported in 96.4% (54/56) and 75% (42/56) of cases, respectively (Table 3). Table 3 also documents the changes in the prevalence of all audiological symptoms one year after diagnosis. Among the different diagnostic assessments, delayed 3D-FLAIR MRI showed the highest pathological findings, observed in 76.8% (43/56) of patients, followed by infectious disease screening, which revealed abnormalities in 21.4% (12/56) of cases (Figure 1). Elevation of liver serum markers was observed in five patients (three diagnosed with EBV infection and two with HSV-1 infection), while no pathological variants in genes associated with HL were detected by NGS testing (Figure 1). In 25% of the patients, the etiology was idiopathic. In the remaining cases, the causes were identified as otological (39.3%), infectious (21.4%), autoimmune (7.1%), vascular (5.4%), and neoplastic (1.8%) (Table 4). In terms of the presence of any type of hearing improvement ascertained one year later, no significant differences were observed between males and females (*p* = 0.25), nor between age groups (*p* = 0.99). Conversely, a significant difference was found when comparing the various causes of SSNHL (*p* = 0.009) (Table 5). In particular, the specific etiologies of patients with improved HT were as follows: isolated cochlear endolymphatic hydrops (n = 3), Menière’s disease (n = 3), primary antiphospholipid syndrome (n = 3), cell disease (n = 2), HSV-1 (n = 1), and SARS-CoV-2 (n = 1). Table 5 also demonstrates that the prevalence of severe-to-profound HL was similar across different sexes (*p* = 0.11), age groups (*p* = 0.75), and causes of HL (*p* = 0.09).

Among the 45 (80.4%) patients who presented with HL at T1 (44 unilaterally and 1 bilaterally), 23 (51.1%) wore a conventional hearing aid (22 unilaterally and 1 bilaterally), 1 (2.2%) a contralateral-routing-of-signal (CROS) device, and 1 (2.2%) a cochlear implant, while 20 (44.4%) decided not to use any hearing device. The reasons for not using a hearing device were aesthetic issues (12/20, 60%), no benefit after a trial period (3/20, 15%), no perceived need (3/20, 15%), and excessive cost (2/20, 10%).

## 4. Discussion

The present study conducted a comprehensive investigation of the hearing characteristics and etiology of SSNHL in patients under 40 years of age who were assessed at a tertiary-level audiologic center. The results confirm that the hearing features of SSNHL are heterogeneous [8,14,15,16], as evidenced by the wide spectrum of HL severity (from mild to profound), audiometric patterns (flat, sloping, or rising configurations), and audiological outcomes (from absence to complete hearing recovery) found in our patients. Following extensive audiological, vestibular, serological, and neuroradiological examinations, a specific cause of SSNHL was not identified in only one out of four patients. This proportion of idiopathic cases is much lower than typically reported in the literature [1,2,17], likely due to the early and thorough diagnostic approach employed in our study. Bilateral SSNHL was observed in only one subject, thereby reinforcing the notion that SSNHL predominantly affects a single ear, especially in younger patients [1,2,18]. The patient who experienced bilateral SSNHL also had bilateral peripheral vestibular hypofunction and was subsequently diagnosed with Cogan’s syndrome. Cogan’s syndrome, a rare autoimmune disease primarily affecting young adults, is marked by non-syphilitic interstitial keratitis and audio-vestibular symptoms; in particular, SSNHL, irrespective of its association with vestibular symptoms, can be the initial manifestation of this syndrome [19]. Although some authors have suggested that younger age is a favorable prognostic factor for SSNHL [20,21,22,23], our data showed that only one in five patients had complete recovery of the HT. In detail, this study revealed that factors such as age and sex did not have a significant impact on the likelihood of having HT improvement or severe-to-profound HL at the audiological evaluation after one year. However, the patients with SSNHL due to neoplastic, infectious, and otological causes demonstrated the lowest frequency of HT improvement over time, highlighting the impact that the underlying cause of SSNHL may have on prospects for hearing recovery [21]. Interestingly, MRIs acquired within the first two weeks after the diagnosis of SSNHL and supplemented with 4 h delayed postcontrast 3D-FLAIR sequences were abnormal in almost four out of five patients; in most cases, these revealed an endolymphatic hydrops or an inflammatory pattern. Specifically, more than 80% of known causes of SSNHL in our study were represented by otological and infectious disorders. Among patients with endolymphatic hydrops detected on MRI, two-thirds subsequently received a diagnosis of definite Menière’s disease [24], while one-third were classified as isolated cochlear endolymphatic hydrops [25], which may explain the high percentage of hearing fluctuations observed during the audiological follow-ups. High-resolution delayed contrast MRI can therefore be a useful diagnostic tool to identify endolymphatic hydrops even in patients with low-to-medium frequency SSNHL who will not meet the criteria for Menière’s disease [24], consequently enabling more appropriate management and treatments [25,26,27]. In addition, MRI of the brain was pivotal in identifying a case of vestibular schwannoma among our patients with SSNHL; this occurrence is in line with the prevalence outlined in the existing scientific literature [28]. It is noteworthy that more than 40% of our patients initially reported experiencing vertigo, which was mainly related to conditions such as Menière’s disease or unilateral acute peripheral vestibular hypofunction; these findings corroborate the need for in-depth vestibular assessments in all cases of SSNHL [29]. However, aural fullness, tinnitus, and hyperacusis emerged as the predominant symptoms associated with SSNHL, and consistent with other studies, they showed a significant decrease in prevalence over time [8,30,31]. Although high-resolution CBCT yielded negative results in most of our patients with SSNHL, it played a crucial role in the diagnosis of otosclerosis and superior semicircular canal dehiscence, even in the absence of typical clinical and audiological characteristics. As a matter of fact, these are well-known causes of SSNHL that should always be considered among the differential diagnoses [1,32]. In our series, CBCT scanning also detected an enlarged vestibular aqueduct in two patients; however, MRI had already thoroughly visualized the malformation in these cases. Notably, this study strengthened the evidence that infectious agents, particularly viruses of the Herpesviridae family and SARS-CoV-2, contribute to the occurrence of a nonnegligible number of cases of SSNHL in adults [33,34], as was previously widely described for the pediatric population [35,36,37,38]. These viruses were also found to be the most common known cause of labyrinthitis, confirming the main etiopathogenetic hypotheses in the current literature [38,39]. Viral infections might induce SSNHL through different mechanisms within the inner ear, such as direct invasion, reactivation of a latent virus, immune-mediated injuries, or abnormal stress responses [34,40]. Furthermore, one patient tested positive for acute Borrelia burgdorferi infection, underscoring the potential relationship between the bacterium that causes Lyme disease and SSNHL.

Indeed, this bacterial infection, which is transmitted to humans by tick bites, could lead to the formation of inflammatory and angiopathic lesions in the cochlear vessels, thus contributing to the onset of SSNHL [41]. Our data emphasized the need for individuals presenting with SSNHL, especially women of reproductive age with a history of vascular thrombosis and/or pregnancy loss, to be tested for anti-β-2-glycoprotein-1 antibodies, lupus anticoagulant, and anti-cardiolipin antibodies; this recommendation stems from the potential role of antiphospholipid syndrome in the pathogenesis of SSNHL, as a consequence of microthrombus formation in the labyrinthine vasculature [42,43]. The present study also confirmed that sickle cell disease, a genetic disorder characterized by abnormal hemoglobin production, is another possible cause of SSNHL in individuals younger than 40 years of age [44,45]. Indeed, these patients have sickle-shaped red blood cells that can obstruct small blood vessels in the inner ear, leading to ischemia and subsequent SSNHL; in addition, the breakdown of sickle cells releases hemoglobin, which can be toxic to the cochlear structures [46]. Similarly, antithrombin III deficiency may predispose patients to abnormal blood clot formation, which probably caused the ischemic cochlear damage underlying the onset of SSNHL observed in one of our patients; however, the relationship between antithrombin III deficiency and SSNHL is still uncertain [47]. If the HT is not fully recovered after treatment, especially in the presence of persistent tinnitus, it may be advantageous to consider the use of hearing aids or, in cases of severe-to-profound HL, cochlear implantation [2,48]. Indeed, individuals with unilateral sensorineural HL often struggle with speech perception in noisy environments and sound localization, which can lead to increased fatigue and restlessness, as well as reduced selective attention and overall quality of life [9,49]. However, it is interesting to note that many patients did not use a hearing aid for purely aesthetic reasons, highlighting the need to strengthen the shared decision-making approach and bolster psychosocial support in the management of patients with SSNHL [2]. The case of the renowned Spanish artist Francisco Goya is a notable example of how SSNHL can influence an individual’s life and work. In November 1792, in Seville, Goya was struck by a serious illness that made him severely hearing impaired and altered his artistic perception. Before that time, Goya’s works, such as “The Meadow of San Isidro” (1788), were characterized by a more traditional and elegant style. Nevertheless, after the onset of SSNHL, his work began to delve into darker and more somber themes. His later works, such as “A Pilgrimage to San Isidro” (1820–23), are an emblem of the artistic change; this painting, part of the series known as the Black Paintings, portrays a collection of distorted figures in the darkness, reflecting the artist’s deep unease that arose from SSNHL [50].

### Limitations and Future Prospects

Although the study contributes to a greater understanding of the characteristics of SSNHL in patients younger than 40 years old, it is essential to acknowledge its limitations. Firstly, the study adopted a retrospective design, which relied on the analysis of medical records that were initially curated for clinical purposes rather than research data collection. This approach led to the omission of some information, such as the exact values of the screening test results (in our database they were reported as normal/pathological dichotomous variables), detailed data on the period between the first month and the twelfth month after diagnosis of SSNHL, and the specific investigations and treatments performed by the specialist physicians to whom the patients were referred. However, it is worth noting that the primary aim of this study was to examine the hearing characteristics and causes of SSNHL in patients aged from 15 to 40 years, with a particular focus on audiological outcomes one year post-diagnosis. Another important limitation is represented by the relatively small group of patients with SSNHL, all from a single audiology center, which may not be representative of a larger population. Considering these factors, it is crucial to conduct additional studies across various centers, involving a substantial number of patients with SSNHL under 40 years of age, to validate and strengthen our findings. Moreover, a prospective comparative study of the causes of SSNHL in subjects younger than 40 years of age versus those older than 40 years of age could lead to the development of age-specific treatment plans and prevention strategies. It would indeed be intriguing to undertake a detailed assessment of how specific treatments, tailored to different causes, could influence the chances of improving hearing. Finally, since the observation period was limited to only one year, a potential improvement for future research could involve lengthening the follow-up period to evaluate long-term audiological outcomes according to different etiologies.

## 5. Conclusions

This study, to the best of our knowledge, is the first to specifically evaluate the characteristics and causes of SSNHL in a patient population aged from 15 to 40 years. A thorough diagnostic investigation, using high-resolution delayed 3D-FLAIR MRI, CBCT, and screenings for coagulation, infectious, and autoimmune diseases, together with timely referrals to the most suitable specialist physician when diagnostic tests showed abnormalities, enabled the identification of the cause of SSNHL in a large percentage of patients under 40 years of age. In this age group, the predominant causes of SSNHL were found to be otological and infectious, with Menière’s disease, isolated cochlear endolymphatic hydrops, HSV-1, and EBV infections being the most frequently observed. The patients with SSNHL evaluated in this study showed high variability spanning across several aspects, such as the HT, audiometric configurations, associated symptoms, hearing changes over time, and adherence to treatment. Identifying the cause responsible for SSNHL in each patient is of paramount importance because it paves the way for a more personalized approach to management and treatment. This could potentially reduce the risk of recurrence of SSNHL and improve long-term outcomes.

## Figures and Tables

**Table 1:** Hearing characteristics of patients with sudden sensorineural hearing loss at the time of diagnosis (T0) and at the 12-month audiological assessment (T12).

Variables	Patients (T0), N (%)	Patients (T12), N (%)
Age		
15–23 years	15 (26.8)	/
24–32 years	23 (41.1)	/
33–40 years	18 (32.1)	/
Side of hearing loss		
None	0 (0.0)	11 (19.6)
Right	24 (42.9)	17 (30.4)
Left	31 (55.4)	27 (48.2)
Bilateral	1 (1.8)	1 (1.8)
Hearing status		
Normal	0 (0.0)	11 (19.6)
Mild hearing loss	10 (17.9)	10 (17.9)
Moderate hearing loss	30 (53.6)	20 (35.7)
Severe hearing loss	10 (17.9)	11 (19.6)
Profound hearing loss	6 (10.7)	4 (7.1)
Audiometric configuration		
Rising	16 (28.6)	12 (21.4)
Flat	20 (35.7)	28 (50.0)
Sloping	20 (35.7)	16 (28.6)
Hearing recovery		
Complete	/	11 (19.6)
Partial	/	10 (17.9)
Slight	/	2 (3.6)
None	/	33 (58.9)
TOTAL	56 (100.0)	56 (100.0)

**Table 2:** Neuroradiological and vestibular examinations in the study population.

Variables	Patients, N (%)
MRI of the brain (including 4 h delayed 3D-FLAIR sequences) within 2 weeks from diagnosis	
Normal	13 (23.2)
Pathological lesions/anatomical abnormalities	4 (7.1)
Hemorrhagic pattern	6 (10.7)
Inflammatory pattern	13 (23.2)
Endolymphatic hydrops	18 (32.1)
Isolated blood–labyrinth barrier breakdown	2 (3.6)
High-resolution cone beam CT of the ear within 2 weeks from diagnosis	
Normal	52 (92.9)
Pathological lesions/anatomical abnormalities	4 (7.1)
Vestibular examination (bedside + v-HIT) at the time of diagnosis	
Normal	48 (85.7)
Unilateral peripheral vestibular hypofunction	7 (12.5)
Bilateral peripheral vestibular hypofunction	1 (1.8)
TOTAL	56 (100.0)

CT = computed tomography; MRI = magnetic resonance imaging; v-HIT = video head impulse test.

**Table 3:** Audiological symptoms associated with sudden sensorineural hearing loss at the time of diagnosis (T0) and one year later (T12).

Variables	Patients (T0), N (%)	Patients (T12), N (%)	*p*-Value
Aural fulness			
Yes	54 (96.4)	37 (66.1)	<0.001
No	2 (3.6)	19 (33.9)	
Tinnitus			
Yes	42 (75.0)	30 (53.6)	0.03
No	14 (25.0)	26 (46.4)	
Hyperacusis			
Yes	33 (58.9)	13 (23.2)	<0.001
No	23 (41.1)	43 (76.8)	
Vertigo			
Yes	23 (41.1)	16 (28.6)	0.23
No	33 (58.9)	40 (71.4)	
Dizziness			
Yes	21 (37.5)	12 (21.4)	0.1
No	35 (62.5)	44 (78.6)	
Otalgia			
Yes	10 (17.9)	2 (3.6)	0.03
No	46 (82.1)	54 (96.4)	
TOTAL	56 (100.0)	56 (100.0)	

**Table 4:** Causes of sudden sensorineural hearing loss in the study population.

Causes	Patients, N (%)
Otological	22 (39.3)
Menière’s disease	12 (21.4)
Isolated cochlear endolymphatic hydrops	6 (10.7)
Isolated enlarged vestibular aqueduct	2 (3.6)
Superior semicircular canal dehiscence	1 (1.8)
Otosclerosis	1 (1.8)
Infectious	12 (21.4)
Herpes Simplex Virus 1	5 (8.9)
Epstein–Barr virus	4 (7.1)
SARS-CoV-2	2 (3.6)
Borrelia burgdorferi	1 (1.8)
Autoimmune	4 (7.1)
Primary antiphospholipid syndrome	3 (5.4)
Cogan’s syndrome	1 (1.8)
Vascular	3 (5.4)
Sickle cell disease	2 (3.6)
Antithrombin III deficiency	1 (1.8)
Neoplastic	1 (1.8)
Vestibular schwannoma	1 (1.8)
Idiopathic	14 (25.0)
TOTAL	56 (100.0)

**Table 5:** Frequency of hearing improvement and prevalence of severe-to-profound hearing loss at the 1-year assessment (T12) according to sex, age, and etiology.

	Total	Hearing Improvement (Evaluated at T12)	*p*-Value	Severe-to-Profound Sensorineural Hearing Loss (Evaluated at T12)	*p*-Value
Sex					
Male	17	9 (52.9)	0.25	2 (11.8)	0.11
Female	39	14 (35.9)		13 (33.3)	
Age					
15–23	15	6 (40.0)	0.99	5 (33.3)	0.75
24–32	23	10 (43.5)		5 (21.7)	
33–40	18	7 (38.9)		5 (27.8)	
Causes of sudden sensorineural hearing loss					
Otological	22	6 (27.3)	0.009	3 (13.6)	0.09
Infectious	12	2 (16.7)		5 (41.7)	
Autoimmune	4	3 (75.0)		1 (25.0)	
Vascular	3	2 (66.7)		2 (66.7)	
Neoplastic	1	0 (0.0)		1 (100.0)	
Idiopathic	14	10 (71.4)		3 (21.4)	
TOTAL	56	23 (41.1)		15 (26.8)	

## Data Availability

The data presented in this study are available on request from the corresponding author.
